# MEK1/2 Inhibition in Murine Heart and Aorta After Oral Administration of Refametinib Supplemented Drinking Water

**DOI:** 10.3389/fphar.2020.01336

**Published:** 2020-08-28

**Authors:** Felke Steijns, Nathalie Bracke, Marjolijn Renard, Julie De Backer, Patrick Sips, Nathan Debunne, Evelien Wynendaele, Frederick Verbeke, Bart De Spiegeleer, Laurence Campens

**Affiliations:** ^1^Center for Medical Genetics, Ghent University, Ghent, Belgium; ^2^Drug Quality and Registration (DruQuar) Group, Faculty of Pharmaceutical Sciences, Ghent University, Ghent, Belgium; ^3^Department of Cardiology, Ghent University Hospital, Ghent, Belgium

**Keywords:** refametinib (RDEA119, BAY 86-9766), RAS-RAF-MEK-ERK-MAPK pathway, murine myocardium, murine aorta, drinking water, UHPLC

## Abstract

Upregulation of the RAS-RAF-MEK-ERK-MAPK pathway is involved in the development of several human tumors, aortic aneurysms, atherosclerosis, and cardiomyopathy. Refametinib, a highly selective MEK-inhibitor, has already shown antineoplastic activity in phase II trials. Furthermore, it showed potency to attenuate aortic root growth in murine models. Current formulations of this drug however necessitate oral gavage as a delivery method for long-term studies, which is labor-intensive and induces stress and occasional injury, potentially confounding results. Therefore, we developed a novel oral administration method for refametinib. A 2-hydroxypropyl-beta-cyclodextrin (HPBCD) based drinking water preparation of refametinib was formulated, for which a selective, analytical UHPLC-UV method was developed to assess the in-use stability. Next, 16 week old male wild-type C57Bl/6J mice received either a daily dose of 50 or 75 mg/kg/day refametinib or were given regular drinking water during 7 days. In both dosage groups the refametinib plasma levels were measured (n = 10 or 7, respectively). Furthermore, pERK/total ERK protein levels were calculated in the myocardial and aortic tissue of mice receiving a daily dose of 50 mg/kg/day refametinib and untreated mice (n = 4/group). After 7 days no significant degradation of refametinib was observed when dissolved in drinking water provided that drinking bottles were protected from UV/visible light. Furthermore, a dose-dependent increase in refametinib plasma levels was found whereby active plasma levels (> 1.2 µg/mL) were obtained even in the lowest dose-group of 50 mg/kg/day. A significant reduction of pERK/total ERK protein levels compared to untreated mice was observed in aortic and myocardial tissue of mice receiving a daily dose of 50 mg/kg/day refametinib. Importantly, a relatively high mortality rate was noted in the highest dose group (n = 5). This approach provides a valid alternative oral administration method for refametinib with a reduced risk of complications due to animal manipulation and without loss of functionality, which can be implemented in future research regarding the malignant upregulation of the RAS-RAF-MEK-ERK-MAPK pathway. However, care must be taken not to exceed the toxic dose.

## Introduction

The MAPK (mitogen-activated protein kinase) pathway is involved in the regulation of a plethora of processes including apoptosis, cell cycle progression, metabolism, cell migration, differentiation and proliferation (Wortzel and Seger, 2011). The pathway consists of different signaling arms, including extracellular signal-regulated kinase (ERK-1 and -2), Jun N-terminal kinase (JNK) and p38 (Derynck and Zhang, 2003). Upstream activators of ERK-1 and -2 are MEK-1 and -2, which are in turn activated by RAS/RAF signaling. And finally, the entire RAS-RAF-MEK-ERK-MAPK signal cascade can be activated through phosphorylation *via* the activation of signaling cascades by an abundance of growth factors and cytokines, including transforming growth factor-β (TGFβ) (Derynck and Zhang, 2003).

Increased or constitutive activation of the RAS-RAF-MEK-ERK-MAPK pathway, either through gain-of-function pathogenic variants in oncogenes or hyperactivation of upstream pathways, has been observed in several human tumors, including lung, colon, melanoma, thyroid, and pancreatic cancer (Grandis and Sok, 2004; Hynes and Lane, 2005; Schubbert et al., 2007; Wang et al., 2007; Murugan et al., 2009). In addition, ERK-1 and -2 activation has also been shown to contribute to aortic aneurysm progression (Holm et al., 2011; Yang et al., 2016), atherosclerosis (Chen et al., 2015; Zhang et al., 2016; Li X. J. et al., 2016) and dilated and hypertrophic cardiomyopathy (Wu X. et al., 2011; Cook et al., 2014; Campens et al., 2015; Li C. et al., 2016; Rouf et al., 2017). It has been reported that inhibition of ERK-1 and -2 phosphorylation increases the elastin synthesis both *in vitro* (in vascular smooth muscle cells) and *in vivo* (in rat aorta) thereby highlighting ERK-1 and -2 inhibition as a potential treatment for vascular pathologies characterized by reduced arterial elastin content (Lannoy et al., 2014). Furthermore, MEK-inhibition also has antiatherogenic properties as MEK-inhibition combined with activation of liver X receptor (LXR) significantly inhibited the development of atherosclerosis in ApoE deficient (ApoE^-/-^) mice through reverse cholesterol transport and by blocking the formation of foam cells (Chen et al., 2015; Li X. J. et al., 2016; Zhang et al., 2016). In a transgenic rabbit model for human hypertrophic cardiomyopathy (β-MHC-Q^403^) MEK-inhibition mitigated the cardiac hypertrophic phenotype and improved the cardiac function (Patel et al., 2001). Furthermore, treatment of *Lmna^H222P/H222P^* mice, known to develop dilated cardiomyopathy, with ERK inhibitors prevented left ventricular dilatation, decreased myocardial fibrosis and blocked increased RNA expression of natriuretic peptide precursors (Wu W. et al., 2011). In addition, MEK-inhibition prevented enlargement of the cardiac myocytes and fetal gene reactivation in an *in vitro* model for cardiac hypertrophy (Li C. et al., 2016). Altogether, these preclinical studies encourage the use of MEK-inhibitors as a strategy to target vascular pathologies and cardiomyopathy caused by an aberrant activation of the RAS-RAF-MEK-ERK-MAPK pathway.

Refametinib (also known as RDEA119 or BAY 86-9766) is a known highly selective, potent and allosteric inhibitor of MEK-1 and -2 (Iverson et al., 2009). Compared to first generation MEK inhibitors, refametinib has been shown to have reduced activity in the brain and a modest cardiovascular toxicity with solely sporadic prolongations of the QT-intervals and reduced ventricular ejection fractions in a small subgroup of cancer patients (Allen et al., 2003; Rinehart et al., 2004; LoRusso et al., 2005; Brown et al., 2007; Weekes et al., 2013). Refametinib is therefore an attractive treatment option for the abovementioned ERK-associated malignant manifestations. Currently, several reports have shown that the compound (either alone or in combination with other therapeutic compounds) has both *in vitro* and *in vivo* antitumor activity in multiple preclinical cancer models by inducing cell cycle arrest or apoptosis (Chang et al., 2010; Schmieder et al., 2013; Dilly et al., 2015; Troiani et al., 2015). Clinical trials for various solid tumors have already proven the positive effect of MEK inhibition on tumor growth (Weekes et al., 2013; Lim et al., 2014; Kiessling and Rogler, 2015; Van Laethem et al., 2017). Refametinib has also been used successfully in a mouse model for Marfan syndrome (MFS, *Fbn1^C1039G/+^*), a syndrome associated with aortic aneurysm, attenuating aortic root growth after two months of treatment compared to placebo-treated mice (Holm et al., 2011). In addition, refametinib treatment also ameliorated aortic disease (decreasing aortic diameters and preventing intimal-medial tears at an early stage of disease) in smooth muscle cell (SMC)-specific *Tgfbr1* deleted (*Tgfbr1^iko^*) mice (Yang et al., 2016). Finally, exacerbated aortic aneurysms and premature death observed in MFS mice treated with Ca^2+^-channel blockers could also be rescued upon refametinib treatment (Doyle et al., 2015).

The current standard dosing strategy for refametinib in preclinical animal trials consists of a twice-daily oral gavage for the duration of the treatment period. However, oral gavage induces significant stress and occasional injury in rodents which can contribute to confounding results by dosing-induced stress even when an appropriate sham group is included in the study (Brown et al., 2000; Balcombe et al., 2004; Bonnichsen et al., 2005; Vandenberg et al., 2014). Based on the 3 Rs principle (*i.e.* reduction, replacement and refinement), alternative methods for oral gavage have been investigated to alleviate the suffering and distress experienced by laboratory animals (Atcha et al., 2010; Vandenberg et al., 2014), such as administration *via* drinking water or food.

To the best of our knowledge, no alternative stress-free routes for administration (*e.g. via* drinking water) are currently explored or available for administering refametinib to laboratory animals. Refametinib is water insoluble and typically administered *via* oral gavage using 2-hydroxypropyl-beta-cyclodextrin (HPBCD) (Holm et al., 2011) and cremophor EL, a surfactant in saline (Chang et al., 2010), as vehicle. As such, we focused our efforts on the evaluation of a suitable drinking water formulation by the development of an analytical method to evaluate the stability for this novel formulation in a preclinical setting. Furthermore, we evaluated the pharmacodynamics/-kinetics of refametinib supplemented drinking water in wild-type C57Bl/6J mice. We assessed the refametinib plasma concentrations as well as the ERK inhibitory potential of refametinib in murine myocardial and aortic tissue after 7 days of treatment.

## Materials and Methods

### Chemicals and Reagents

Refametinib (chemical formula: (S)-N-(3,4-difluoro-2-(2-fluoro-4-iodophenylamino)-6-methoxyphenyl)-1-(2,3-dihydroxypropyl) cyclopropane-1-sulfonamide, > 99% chemical and optical purity) was purchased from ChemieTek (Indianapolis, IN, USA). Acetonitrile and methanol of ultra-high performance liquid chromatography (UHPL)-gradient grade were obtained from Fisher Scientific (Merelbeke, Belgium). Water for UHPLC was purified by an Arium 611 purification system (Sartorius, Göttingen, Germany), yielding ≥ 18.2 MΩ x cm quality water. Formic acid (UHPLC/MS grade) was purchased from Fisher Scientific (Merelbeke, Belgium), hydrogen peroxide 30% w/w was from Merck (Overijse, Belgium) and 2-hydroxypropyl-β-cyclodextrin (HPBCD, kleptose, oral grade, concentration/purity ≥ 97%) was from Roquette (Lestrem, France). All other chemicals were from Sigma Aldrich (Overijse, Belgium) and HPLC vials were from Waters (Zellik, Belgium).

### Preparation of Refametinib Drinking Water Formulation

Refametinib was complexed with HPBCD as previously described (Bouquet et al., 2007) with minor adaptations. The desired amount of refametinib was dissolved in absolute ethanol in a ratio of 1/60 w/w. HPBCD was added to the solution to obtain a refametinib/HPBCD ratio of 1/60 mol/mol and the solution was placed in an ultrasonic bath for 5 min. More absolute ethanol was added to obtain a final refametinib/ethanol ratio of 1/223 w/w. Next, phosphate-buffered saline (PBS) was added in a ratio of 2/1 w/w (PBS/HPBCD). Then, the solution was aliquoted in vials and freeze dried (program described in **Supplemental Table S1**) in a Christ gamma 1-16 LSC freeze dryer (Analis s.a.-n.v., Suarlee, Belgium) to obtain a white amorphous powder. After lyophilisation, the refametinib containing vials were stored at −35°C and protected from light.

### UHPLC Method

In order to evaluate the inclusion procedure and to measure the amount of refametinib that was complexed with HPBCD, an amount of freeze dried material was dissolved in tap water to a final concentration of 0.1 mg refametinib/mL, followed by centrifugation (20,000 g for 30 min) and filtration (Whatman^®^ qualitative filter paper Grade 1). In addition, a refametinib standard was prepared in 10% methanol in water (regarded as the 100% concentration-value). The concentration of dissolved refametinib in all samples was determined using an adjusted UHPLC-UV method.

Chromatography was performed using a Water Alliance UHPLC (Waters Corporation, Zellik, Belgium; 375 µL dwell volume) consisting of an autosampler, quaternary pump and Waters Empower chromatographic software. The sample compartment was kept at 5°C (± 2°C), the column was a 100 mm x 2.1 mm, 1.7 µm Acquity UHPLC BEH C_18_ column (Waters Corporation, Zellik, Belgium) held at 40°C (± 5°C), applying a constant flow rate of 0.5 mL/min and using an injection volume of 2.0 µL. Mobile phases were 80/20 (V/V) H_2_O/acetonitrile (ACN) supplemented with 0.1% formic acid (FA) (W/V) (mobile phase A) and 90%/10% ACN/H_2_O supplemented with 0.1% FA (W/V) (mobile phase B). The gradient elution was as follows: mobile phase B was held at 0% during the first 1 min, then increased to 100% in 11 min and held at 100% for 1 min. Then mobile phase B was brought back to 0% in 1 min and held to the original condition for 4 min to enable equilibration of the start conditions, yielding a total run time of 18 min.

Detection was performed using a Waters Photo Diode Array (PDA) Detector (Waters Corporation, Zellik, Belgium) between 190 and 400 nm, with quantification at 230 nm. A reporting threshold of 0.05% of the main peak (i.e. the compound of interest) was applied.

Mass spectrometry for identification of degradants was performed on a Waters Acquity UPLC-MS/MS system, the Synapt G2-Si, (Waters Corporation, Zellik, Belgium) operating in positive ion mode. The nebulization gas was set to 7.00 bar, the desolvation gas flow at 1000 L/h at a temperature of 500°C, the cone gas set to 150 L/h, and the source temperature set to 150°C. A capillary voltage and a cone voltage were set to 3.00 kV and 20 V, respectively. The MS scan mode was set to 50-800 m/z. Argon was employed as the collision gas at 0.16 mL/min. The molecular formula was predicted using a recursive algorithm as previously described (Patiny and Borel, 2013).

### Refametinib Stability Study

In order to analyze the stability of refametinib dissolved in drinking water under different commonly observed conditions in animal facilities, the lyophilised refametinib/HPBCD complex was dissolved in tap water to obtain a final concentration of 0.26 mg refametinib/mL and sonicated for 40 min followed by filtration as previously described. Next, the refametinib solution was divided over the different recipients as follows:

For the adsorption study, three 250 mL drinking bottles were filled with 40 mL of refametinib solution and stored horizontal, three 10 mL volumetric glass flasks were filled with 10 mL refametinib solution and stoppered and additionally, three 10 mL volumetric flasks were filled with placebo (i.e. 38 mg/mL HPBCD in tap water) and stoppered. Both the drinking bottles as the volumetric flasks were protected from UV/VIS light by means of aluminum foil wrapping. Subsequently, the drinking bottles and volumetric glass flasks were either stored at 5°C, 25°C/60% relative humidity (RH) or 40°C/75% RH.

For the light stability study, three drinking bottles, of which one was wrapped in aluminum foil (= control, protected from light), were filled with 40 mL of refametinib solution and placed horizontal for storage in a UV and visible light chamber at 25 ± 2°C and 60 ± 5% RH.

For the in-use study, 40 mL of refametinib solution was delivered to the mouse facility at the Ghent University Hospital (average minimum-maximum temperature and relative humidity: 19–24°C and 41–59% RH) and placed in a drinking bottle wrapped in aluminum foil.

Next, for all abovementioned studies, samples were taken immediately (t_0_) and at 7 days (t_7_) for further UHPLC analysis. The percentage refametinib recovery at t_7_ was calculated, relative to the percentage refametinib recovery at t_0_ and chromatograms were analysed for potential degradation peaks.

### Mice

The 16 weeks old male wild-type C57Bl/6J mice were kept in accordance with the institutional guidelines regarding the care, housing and use of laboratory animals, which are based on the European Parliament Directive 2010/63/EU. The procedure was approved by the Ethics Committee for the care and use of laboratory animals of the Ghent University Hospital (EC 15/77).

### Pharmacological Treatment and Sample Collection Mice

Based on previously recorded average daily drinking volume in wild-type C57Bl/6J mice (see **Supplemental Table S2**) a sufficient amount of lyophilized refametinib/HPBCD complex was dissolved in drinking water as previously described in *Section 2.2* in order to obtain refametinib concentrations of 50 or 75 mg/kg/day. These concentrations were based on the study of Iverson et al. (2009) as they reported that an active refametinib plasma concentration of at least 1.2 µg/mL was reached after twice-daily administration of 25 mg/kg. The additional higher dosage of 75 mg/kg/day refametinib was also included in order to buffer potential variations in water uptake, even though drinking behavior was not influenced by addition of refametinib when evaluated prior to initiation of treatment.

Drinking bottles were protected from light with aluminum foil and the volume was carefully documented. Next, a total of 16 mice received 50 mg/kg/day refametinib (group 1), 12 mice received 75 mg/kg/day refametinib (group 2), and 4 mice were given regular drinking water (group 3). After 7 days of treatment approximately 200 μL blood was collected from the tail vein in EDTA tubes (BD Microtainer K2E, BD Biosciences), for plasma bioanalyses (subgroup of group 1, n = 10, and all surviving mice of group 2, n = 7). Blood collection was performed at different time points (morning, noon and evening) and executed once in each individual mouse in order to evaluate the stability of the refametinib plasma concentration throughout the entire day. This procedure was performed under general anesthesia (1–1.5% isoflurane mixed with 0.5 L/min 100% O_2_). Subsequently, the blood samples were centrifuged for 10 min at 1,000–2,000 g and 4°C. Plasma was aliquoted into new tubes and stored at −80°C prior to analysis.

### Plasma Bioanalysis

Plasma samples were analyzed for refametinib using a UHPLC-tandem mass spectrometry (UHPLC-MS/MS) method. Prior to analysis, samples were diluted 1/25 in water and vortexed for 5 s. Subsequently, 50 µL was taken for the sample preparation procedure adapted from Iverson et al. (2009). In short, 200 µL of the internal standard (20 ng/mL) dissolved in 1% formic acid/5/95 V/V acetonitrile/H_2_O was added to the sample and vortexed for 15 s. Next, samples were centrifuged for 15 min at 20,000 g at room temperature. Finally, the supernatant was collected and analyzed by means of UHPLC-MS/MS in order to obtain the refametinib plasma concentration.

### Protein Expression Studies

Mice receiving 50 mg/kg/day refametinib (no blood draw performed) and untreated mice were sacrificed (n = 4/group) by means of CO_2_ overdose (1.0 L/min). Hearts and aortae were flushed *in situ* with 1x phosphate buffered saline (PBS) and dissected. Tissue samples were kept on ice and homogenized by mixing or crushing in lysis buffer (RIPA, Sigma-Aldrich) complemented with protease inhibitors (Complete protease inhibitor cocktail tablets, Roche) and phosphatase inhibitors (Cocktail II and III, Sigma-Aldrich) at a 30% (W/V) ratio. Lysates were centrifuged at 20,000 g at 4°C for 30 min and supernatants were collected and stored at −80°C until further processing. Total protein concentration of the samples was determined using the Pierce BCA (bicinchoninic acid) 660 nm Protein assay kit (Thermo Fisher Scientific). Protein samples (50 µg) diluted to a total volume of 20 µL with PBS were reduced by adding 2 µL 1 M dithriothreitol (Sigma-Aldrich) and incubation at 95°C for 5 min. Next, samples were loaded on a NuPage 4–12% Bis-Tris gel (Invitrogen) together with 5x non-reducing lane marker sample buffer (Thermo Fisher Scientific). Following SDS-PAGE electrophoresis, proteins were transferred onto a nitrocellulose membrane (Invitrogen) by means of the iBlot 2 dry blotting system (Thermo Fisher Scientific). The membrane was blocked in 2% bovine serum albumin for one hour and incubated overnight at 4°C with the primary antibody rabbit monoclonal anti-phospho-p44/42 MAPK (pERK) (1:2000, Cell Signalling Technologies). Next, membranes were incubated with a secondary antibody, anti-rabbit IgG HRP-linked (1:5000, Cell Signaling Technologies) for 1 hour at 4°C. Subsequently, membranes were incubated with the SuperSignal West Dura luminol-based ECL HRP substrate (Thermo Fisher Scientific) for 5 min at room temperature. Membranes were scanned using the Chemidoc-it imaging system (UVP, Sopachem). After imaging, the blots membranes were incubated in stripping buffer (Thermo Fisher Scientific) and blocked in 2% ECL (enhanced chemiluminescence)-Advance blocking buffer (GE Healthcare) for 2 h. Subsequently, the entire process was repeated with the primary antibody rabbit polyclonal anti-p44/42 MAPK (ERK) (1:1000, Cell Signaling Technologies). Quantification of the immunoblots was performed using Image J software (v. 1.44p). Statistics GraphPad Prism version 8.0.0 (GraphPad Software, San Diego, California USA) was used for statistical analyses and generating graphs. Independent two-sample t-test was performed on the obtained values of the refametinib stability study, refametinib plasma levels and protein expression studies. Results are shown as mean ± standard error. A p-value of **<** 0.05 was used to define statistical significance (two-sided).

## Results

### Refametinib Stability Study

**Figure 1:**
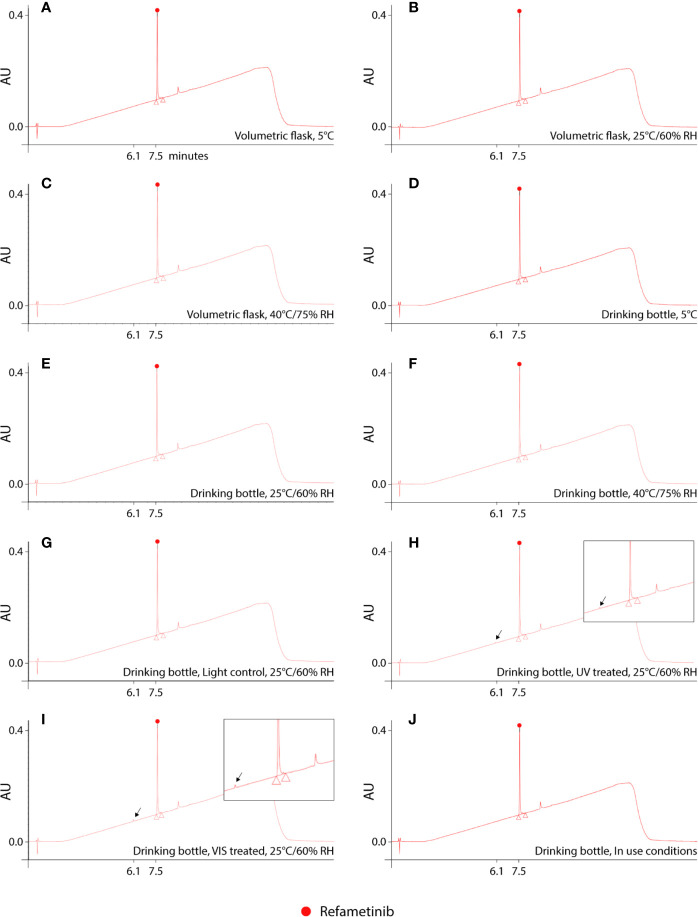
UHPLC chromatograms of dissolved refametinib/HPBCD complex stored for 7 consecutive days under different conditions. **(A–C)** represents the obtained chromatograms of dissolved refametinib/HPBCD complex stored for 7 days in a volumetric flask at 5°C, 25°C/60% RH and 40°C, 75% RH, respectively. No degradation peaks could be observed at any of these conditions. **(D–F)** represents the obtained chromatograms of dissolved refametinib/HPBCD complex stored for 7 days in drinking bottles at 5°C, 25°C/60% RH and 40°C, 75% RH, respectively. The use of drinking bottles did not result in any adsorption of refametinib as no degradation peak could be observed. **(G–I)** representative chromatograms of the content of a control versus treated drinking bottle filled with dissolved refametinib/HPBCD complex and stored in a UV cabinet and VIS cabinet at 25°C/60% RH for 7 consecutive days, respectively. UV-treatment resulted in the appearance of a degradation peak with a retention time of 6.1 min in the chromatogram (indicated with arrow). VIS-treatment resulted in a degradation peak at 6.1 min (indicated with arrow). **(J)** representative chromatogram of dissolved refametinib/HPBCD complex in a drinking bottle stored for 7 days under “in use conditions”. No degradation of refametinib could be observed based on the absence of degradation peaks. AU, absorbance units; RH, relative humidity; UV, ultraviolet light; VIS, visible light.

**Figure 2:**
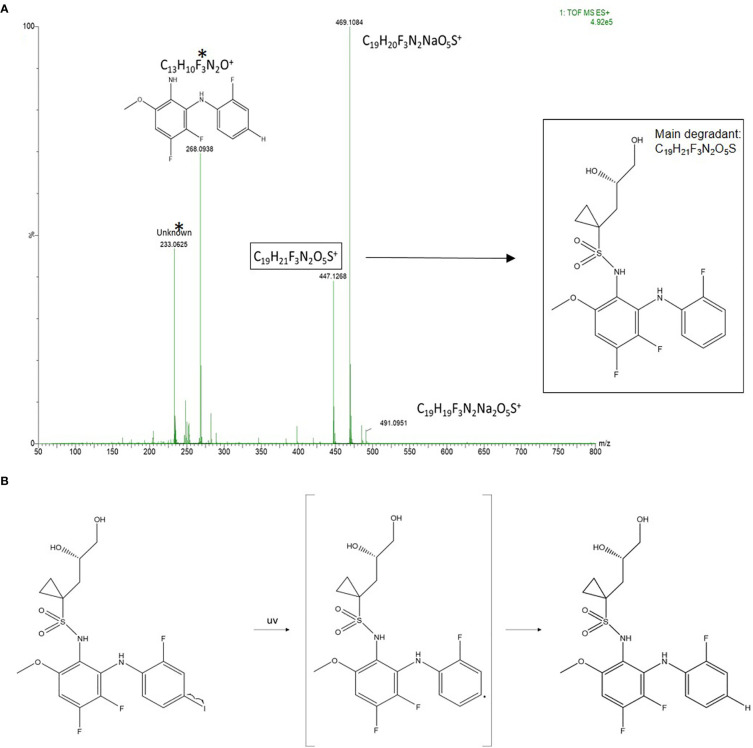
Identification of the main degradant from the VIS light treated samples at t_7_. **(A)** MS-spectra of the refametinib degradant (RRT to refametinib = 0.8). * The peaks with an m/z of 268.0938 and 233.0625 are assigned as potential daughter molecules from the degradant after insource fragmentation. **(B)** The proposed chemical degradation of refametinib to C_19_H_21_F_3_N_2_O_5_S after UV-treatment.

Reconstitution of the lyophilized refametinib/HPBCD complex in tap water, at a ratio of 1/60 mol/mol, resulted in an inclusion efficiency of 100%. Hence, the solubilisation protocol described in *Section 2.2* was used for all following experiments. The results of the stability study are summarized in **Table 1**. Based on the 95% confidence interval on the assay values (i.e. the recoveries) of the controls, degradation of refametinib was considered significant when the recovery would be below 90%. A second criterion, which is more sensitive and discriminatory, is the appearance of degradation peaks in the chromatograms (**Figure 1**). Refametinib was stable in the volumetric glass flasks at all examined conditions, *i.e.* no degradation peaks were observed and an assay recovery (t_0_/t_7_) of > 96% was obtained (**Figures 1A–C**). As for the drinking bottles stored under the same conditions, no significant loss due to adsorption was observed (independent two-sample t-test, p > 0.05) which was confirmed by the absence of degradation peaks on the chromatograms (**Figures 1D–F**). The assay values of the UV light treated refametinib solution at t_7_ amounted to 97%. However, a degradation peak with a retention time (RT) of 6.1 min was observed at t_7_ (0.7% of the refametinib peak area at t_0_), whereas the control sample did not reveal any degradation based on the absence of degradation peaks in the chromatogram (**Figures 1G, H****)**. The assay value of the VIS light treated refametinib compound at t_7_ amounted to 95% and similarly to the UV light treatment, a degradation peak with a RT of 6.1 min was observed at t_7_ (1.4% of the refametinib peak area at t_0_) (**Figure 1I**). The main degradant (relative retention time (RRT) to refametinib = 0.8) was identified as C_19_H_21_F_3_N_2_O_5_S which was confirmed by the experimentally obtained isotopic distribution versus the theoretical distribution, with a high correlation between the relative intensities of the corresponding peaks in both spectra (R² = 0.9996) and the MS/MS-spectra (**Figure 2**). Furthermore, for the in-use conditions, the refametinib assay recovery value was 91% (**Table 1**), i.e. above 90%, and no degradation peak was observed (**Figure 1J**); hence, a sufficient stability under these in use conditions was concluded.

**Table 1:** Summary of the refametinib assay values in volumetric flasks and drinking bottles at t_7_ relative to t_0_ (n = 1).

Container	Conditions	Percentage refametinib recovery at t_7_
Volumetric flask	5°C	99%
	25°C/60% RH	99%
	40°C/75% RH	96%
Drinking bottle	5°C	93%
	25°C/60% RH	93%
	40°C/75% RH	99%
	Light control, 25°C/60% RH	96%
	UV treated, 25°C/60% RH	97%
	VIS treated, 25°C/60% RH	95%
	In use conditions	91%

RH, relative humidity; UV, ultraviolet light; VIS, visible light.

### Dose-Dependent Increase in Refametinib Plasma Levels

**Figure 3:**
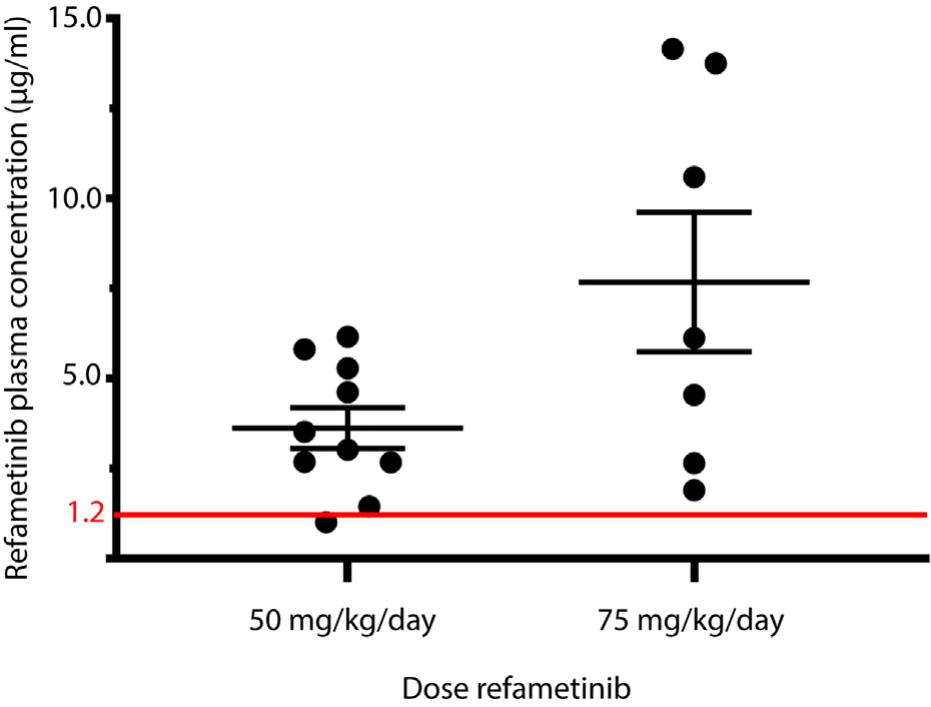
Refametinib plasma levels. Presence of refametinib was observed in the plasma of mice after administration of 50 or 75 mg/kg/day refametinib supplemented drinking water for 7 days. No significant difference in refametinib plasma levels was observed between the different dosage groups (independent two-sample t-test, p = 0.085). Red line at 1.2 µg/mL represents active refametinib serum concentrations previously observed by Iverson et al. (2009).

**Figure 4:**
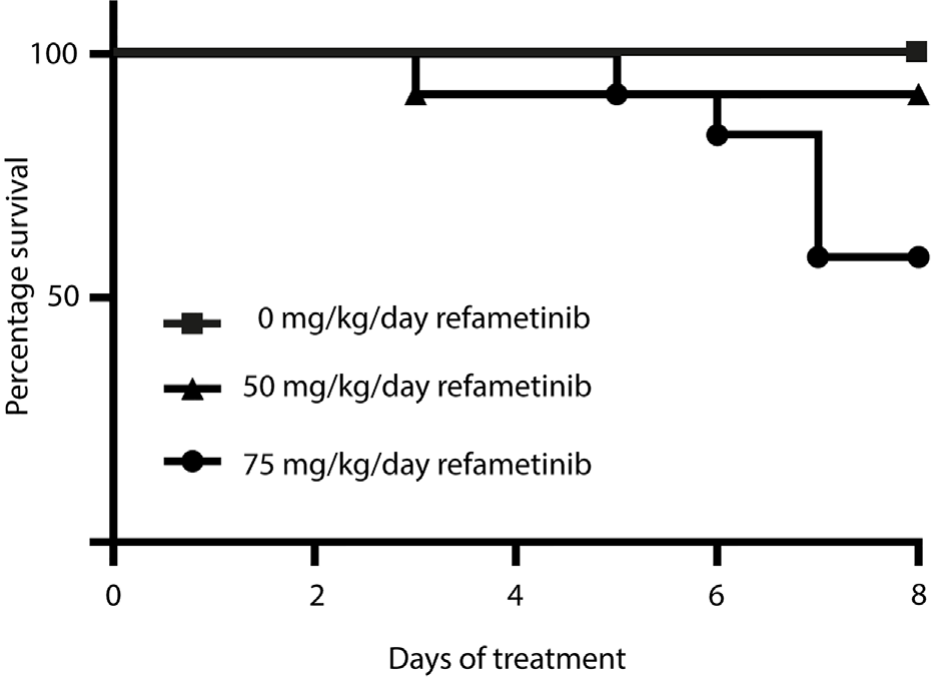
Survival rate within each refametinib dosage group. One mouse out of 16 died during treatment with 50 mg/kg/day refametinib. Five out of 12 mice died during treatment with 75 mg/kg/day refametinib.

After 7 days of refametinib treatment significant plasma levels of refametinib could be observed in both dosage groups, 50 or 75 mg/kg/day refametinib. Furthermore, with increasing dose of refametinib in the drinking water, the plasma levels of refametinib increased (3.62 ± 1.79 μg/mL for 50 mg/kg/day; 7.67 ± 5.14 μg/mL for 75 mg/kg/day), though this increase was not statistically significant (independent two-sample t-test, p = 0.085) (**Figure 3**). In addition, no significant difference in refametinib serum concentration could be observed between the different time points of blood collection within each dosage group (data not shown). With the exception of one mouse, all observed refametinib plasma levels were above 1.2 µg/mL (red line in **Figure 3**), which was previously reported to be an active refametinib plasma concentration (Iverson et al., 2009). During refametinib treatment, one out of sixteen mice receiving 50 mg/kg/day refametinib and five out of twelve mice receiving 75 mg/kg/day refametinib died (**Figure 4**).

### Refametinib Is a Potent Inhibitor of ERK Activation

**Figure 5:**
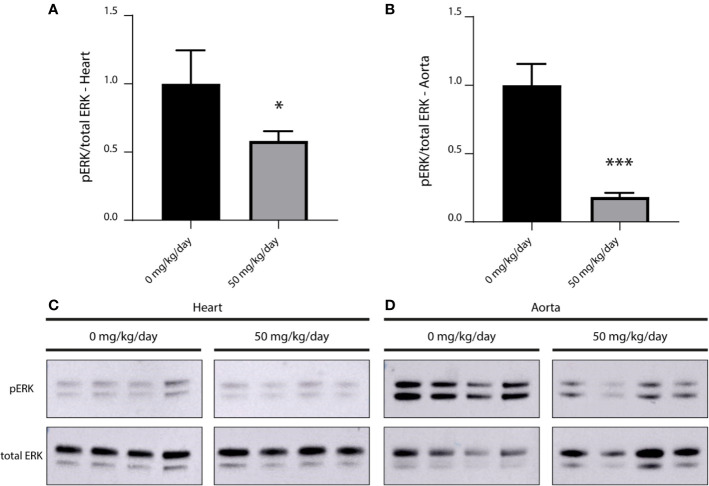
Inhibitory effect of refametinib on ERK activation in murine myocardial and aortic tissue. Refametinib strongly suppressed pERK levels in myocardial **(A)** and aortic **(B)** tissue of mice treated for 7 days with 50 mg/kg/day refametinib supplemented drinking water compared to untreated mice. pERK expression was calculated based on the total ERK expression after which values were normalized to the mean value of untreated mice. Western blots of pERK and total ERK levels in myocardial **(C)** and aortic **(D)** tissue at indicated doses of refametinib supplemented drinking water. Samples represent biological replicates. * independent two-sample t-test, p = 0.0169, *** independent two-sample t-test, p < 0.001.

We compared the level of phosphorylated ERK (pERK) in myocardial and aortic tissue of treated (50 mg/kg/day refametinib) and untreated mice (n = 4/group). Mice receiving 75 mg/kg/day refametinib were not included in this study due to the high death rate previously observed in this dosage group. Administration of refametinib supplemented drinking water strongly suppressed pERK levels in the myocardial and aortic tissue of mice treated for 7 days with refametinib supplemented drinking water compared to untreated mice (a reduction of approximately 42 and 82% respectively, independent two-sample t-test, p ≤ 0.0169) (**Figure 5**).

## Discussion

In the current study we propose an alternative to oral gavage of refametinib, a MEK-1 and -2 inhibitor, which requires less hands-on time, and evokes no stress in laboratory animals. We dissolved refametinib in the drinking water of mice without loss of stability and function based on the observed reduced pERK levels in the murine myocardial and aortic tissue after 7 days of treatment.

We report that HPBCD has a high affinity for refametinib, thereby inducing a significant increase of refametinib solubility, which allowed the drug to be dissolved in laboratory animal drinking water. Furthermore, a selective gradient UHPLC method was developed to evaluate the stability of refametinib supplemented water during different environmental conditions. Overall, the developed UHPLC method met the selectivity and precision requirements for our purposes as degradation products of refametinib could be clearly separated from refametinib itself.

No adsorption, nor degradation of refametinib could be observed in the drinking bottles stored at different temperatures and RH and during in-use conditions. In contrast, degradation of refametinib was observed in drinking bottles stored for 7 days in UV- and VIS-cabinets, with a higher percentage of degradation products observed in the latter based on the chromatograms. The drinking bottles were made of clear polycarbonate (PC). In general, PC is almost completely transparent throughout the entire visible region until 400 nm, blocking the majority of UV light explaining why degradation is higher in the drinking bottles exposed to VIS. As refametinib contains a sulfonamide moiety it is likely prone to photocatalytic degradation in aqueous solutions (Baran et al., 2006; Garcia-Galan et al., 2008). Photocatalytic cleavage of refametinib might result in the formation of an aromatic amine, which is potentially carcinogenic (Benigni and Bossa, 2006). However, as the main degradation product of refametinib after UV/VIS exposure was identified as C_19_H_21_F_3_N_2_O_5_S there is no evidence for photocatalytic cleavage of the sulfonamide site. Therefore, formation of the carcinogenic aromatic amine is not likely. In conclusion, we have successfully dissolved refametinib in drinking water without any significant adsorption nor degradation provided that drinking bottles are wrapped with aluminum foil to exclude formation of degradation compounds. Furthermore, the described formulation might also offer a novel strategy for administration of other water insoluble compounds.

Next, we demonstrated that refametinib supplemented water uptake for 7 days resulted in a sufficiently high active refametinib plasma concentration in all but one wild-type mice for both doses [> 1.2 μg/mL (Iverson et al., 2009)]. The insufficient refametinib plasma concentration (1.010 µg/ml refametinib) observed in the abovementioned single mouse might be due to a relatively low daily water uptake (2.5 mL, lowest observed water uptake) as a result of stress as cage mates belonged to the morning blood draw group whereas the blood draw of the respective mouse took place in the evening.

Significant mortality was observed in the group receiving the highest dose of 75 mg/kg/day refametinib (5 out of 12, 42%). Although increased or constitutive activation of the RAS-RAF-MEK-ERK-MAPK pathway is involved in several pathophysiological manifestations, this pathway is well known to exert a cytoprotective function during physiological conditions (Widmann et al., 1999; Gallo et al., 2019). In addition, downregulation of the RAS-RAF-MEK-ERK-MAPK pathway has been associated with cardiotoxicity as ERK activation plays an important role in preventing the transition from adaptive hypertrophy to heart failure during pressure overload (Harris et al., 2004; Mutlak et al., 2018; Gallo et al., 2019). The otiose inhibition of the RAS-RAF-MEK-ERK-MAPK pathway as a result of high refametinib plasma concentrations might thus be related to the high death rate in the 75 mg/kg/day dosage group. Furthermore, the high variability in refametinib plasma concentration observed in the remaining surviving mice allocated to the high dosage group might also be related to the negative side effects of excessive RAS-RAF-MEK-ERK-MAPK pathway inhibition resulting in morbidity in these mice with subsequent reduced refametinib supplemented water uptake. Regarding the sole case of mortality observed in the lower dosage group of 50 mg/kg/day it is plausible that this mouse was not in an optimal condition prior to the start of the refametinib treatment considering the time of death at the beginning of the treatment.

Finally, administration of 50 mg/kg/day refametinib supplemented water during 7 days effectively reduced ERK-1 and -2 activation in both myocardial and aortic tissue of wild-type C57Bl/6J mice compared to untreated mice (reduction up to 42 and 82%, respectively). Thus, no loss of function of refametinib was observed as a consequence of the protocol used to dissolve refametinib in the drinking water.

In conclusion, we developed a method to dissolve refametinib in the drinking water of laboratory animals. We showed that mice are willing to drink refametinib supplemented water resulting in active refametinib plasma concentrations which effectively reduced ERK-1 and -2 activation in murine myocardial and aortic tissue. Refametinib dissolved in drinking water offers a novel strategy for MEK-1 and -2 inhibition in future preclinical research. However, high dosages of dissolved refametinib should be avoided as these are associated with toxic side effects.

## Limitations

Since this study was not designed to investigate the toxic effects of refametinib, we did not follow up on the mortality that was observed in the higher dosage group of 75 mg refametinib/kg/day. As observed both in our study and the study of Iverson et al. (2009) the refametinib plasma concentration strongly correlates with the administered dose. The higher toxicity observed could thus be due to the administered dose exceeding the toxic level, which should be explored further in follow-up studies.

## Data Availability Statement

The datasets generated for this study are available on request to the corresponding author.

## Ethics Statement

The animal study was reviewed and approved by Ethische Commissie Dierproeven Faculteit Geneeskunde en Gezondheidswetenschappen Universiteit Gent.

## Author Contributions

JB, MR, and LC: Conceptualization. FS, MR, LC, and NB: Methodology. FS, NB, and ND: Data collection. FS, MR, NB, LC, ND, EW, and FV: Formal analysis and investigation. FS, NB, and MR: Writing—original draft preparation. LC, JB, BS, FV, and PS: Writing—review and editing. LC, JB, and BS: Supervision. All authors contributed to the article and approved the submitted version.

## Funding

FS is funded by the Methusalem Grant from Ghent University (Grant number 08/01M01108). JB and MR are Senior Clinical Researchers and Postdoctoral fellow, respectively, supported by the Scientific Research Fund Flanders. PS was supported by the European Union’s Horizon 2020 research and innovation program, under the Skłodowska-Curie grant agreement No. 794365. ND is supported by the Research Foundation Flanders (Grant number 1S21017N). FV was funded by the ‘Institute for the Promotion of Innovation through Science and Technology in Flanders (IWT-Vlaanderen)’ (Grant number 131356). This study is supported by a Grant of the Canadian Marfan Association (now Genetic Aortic Disorders Association Canada).

## Conflict of Interest

The authors declare that the research was conducted in the absence of any commercial or financial relationships that could be construed as a potential conflict of interest.
