# Supplementary material for: MEK1/2 Inhibition in Murine Heart and Aorta After Oral Administration of Refametinib Supplemented Drinking Water
Source: Front Pharmacol. 2020 Aug 28;11:1336. doi: 10.3389/fphar.2020.01336 (PMC7483920; doi:10.3389/fphar.2020.01336)
Supplement: Supplementary file 1 [file DataSheet_1.docx]

# Supplementary Material

**Supplementary table S1: lyophilisation program for refametinib complexed with HPBCD.**

| **Process step** | **Section time (hours:min)** | **Shelf temperature (°C)** | **Chamber pressure (mbar)** |
| --- | --- | --- | --- |
| 1 | 00:00 | 20 | 1000 |
| 2 | 00:15 | 2 | 1000 |
| 3 | 00:20 | -5 | 1000 |
| 4 | 01:00 | -30 | 1000 |
| 5 | 01:20 | -30 | 1000 |
| 6 | 00:15 | -25 | 1.030 |
| 7 | 03:00 | -25 | 1.030 |
| 8 | 02:00 | -15 | 1.030 |
| 9 | 05:00 | -15 | 1.030 |
| 10 | 05:00 | 0 | 1.030 |
| 11 | 15:00 | 0 | 1.030 |
| 12 | 00:15 | 0 | 0.140 |
| 13 | 01:00 | 10 | 0.140 |
| 14 | 07:00 | 10 | 0.140 |

**Supplementary table S2: average daily drinking volume of wild-type male C57Bl/6J mice.**

|  | **Weekly drinking volume** | **ml/ week/ mouse** | **ml/ day/ mouse** |
| --- | --- | --- | --- |
| 3 males | 101.39 | 33.80 | 4.83 |
| 1 male | 37.89 | 37.89 | 5.41 |
| 1 male | 30.57 | 30.57 | 4.37 |
| 7 males | 225.48 | 32.21 | 4.60 |
| 5 males | 202.78 | 40.56 | 5.79 |
| 2 males | 79.81 | 39.91 | 5.70 |
| 2 males | 56.81 | 28.41 | 4.06 |
| 1 male | 41.24 | 41.24 | 5.89 |
| 3 males | 116.17 | 38.72 | 5.53 |
| 1 male | 56.36 | 56.36 | 8.05 |
|  |  | **Average (ml):** | **5.42 ± 1.12** |


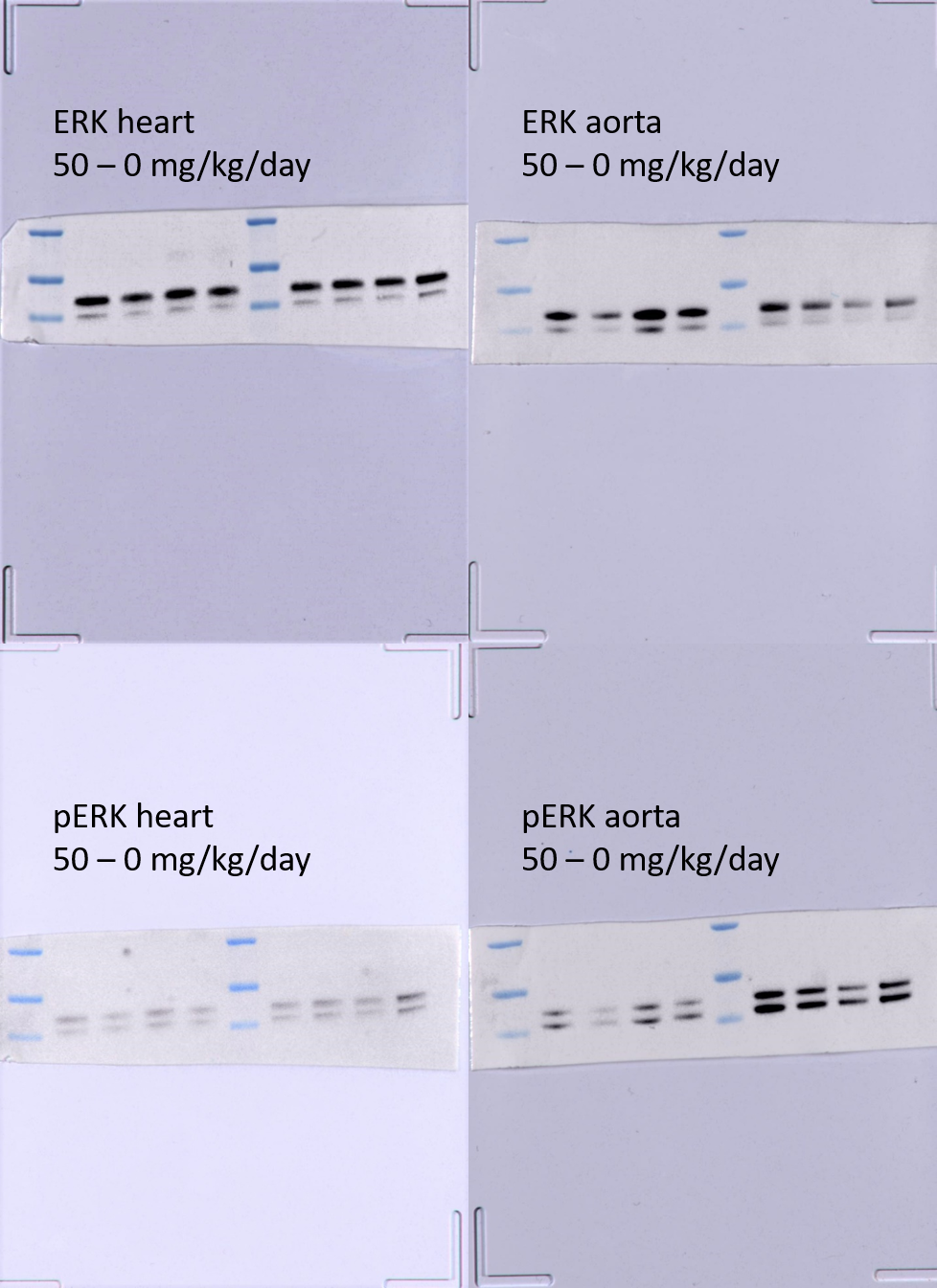


**Supplemental figure S1: Full scan of the original blots of cropped images shown in figure 5.** Lane 1 and 6; ladder. Lane 2-5; 50 mg refametinib/kg/day group. Lane 7-10: control.
