# Supplementary figures and images for: MEK1/2 Inhibition in Murine Heart and Aorta After Oral Administration of Refametinib Supplemented Drinking Water
Source: Front Pharmacol. 2020 Aug 28;11:1336. doi: 10.3389/fphar.2020.01336 (PMC7483920; doi:10.3389/fphar.2020.01336)

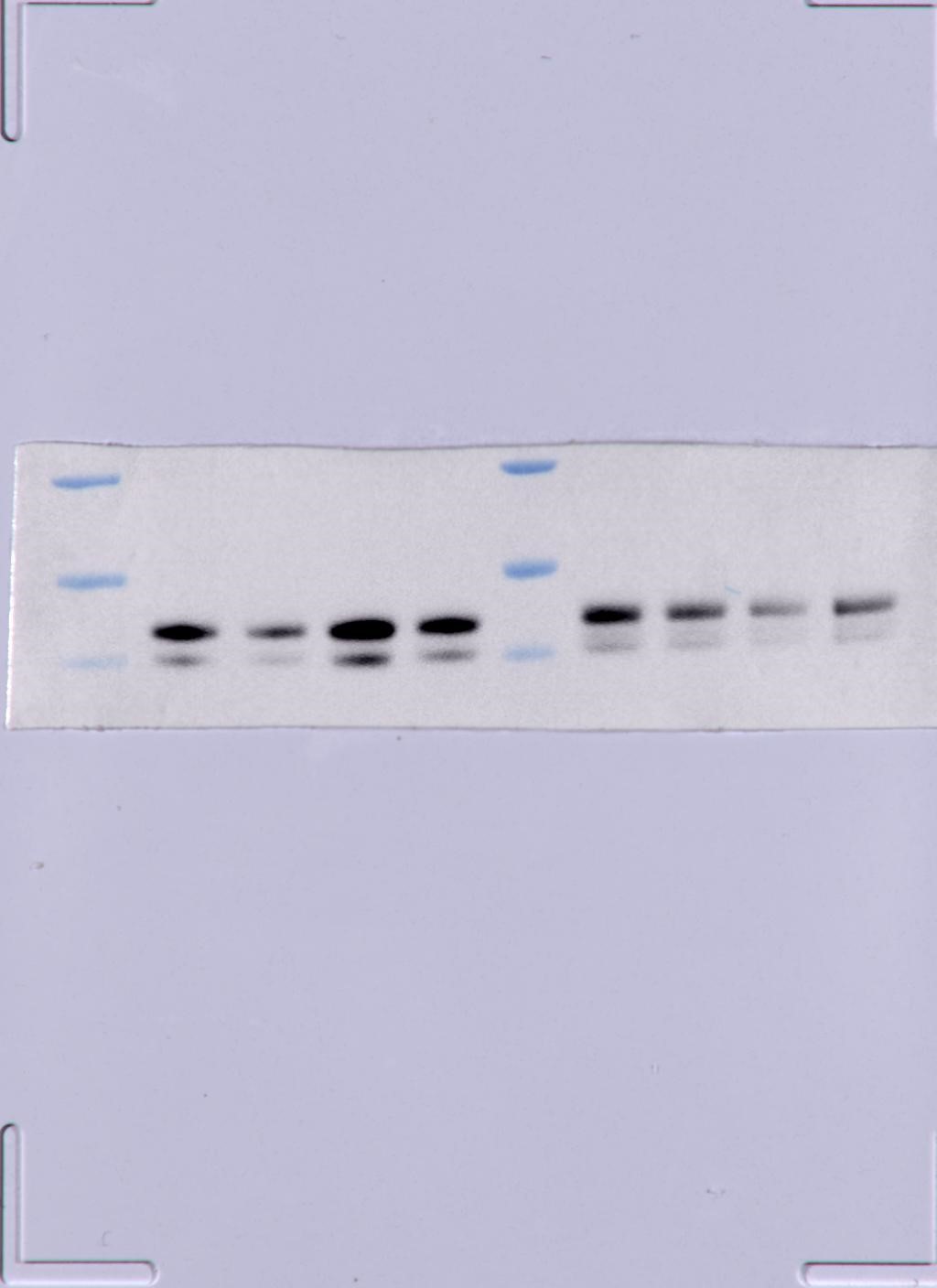

Supplement: Supplementary file 2 [file Image_1.jpeg]

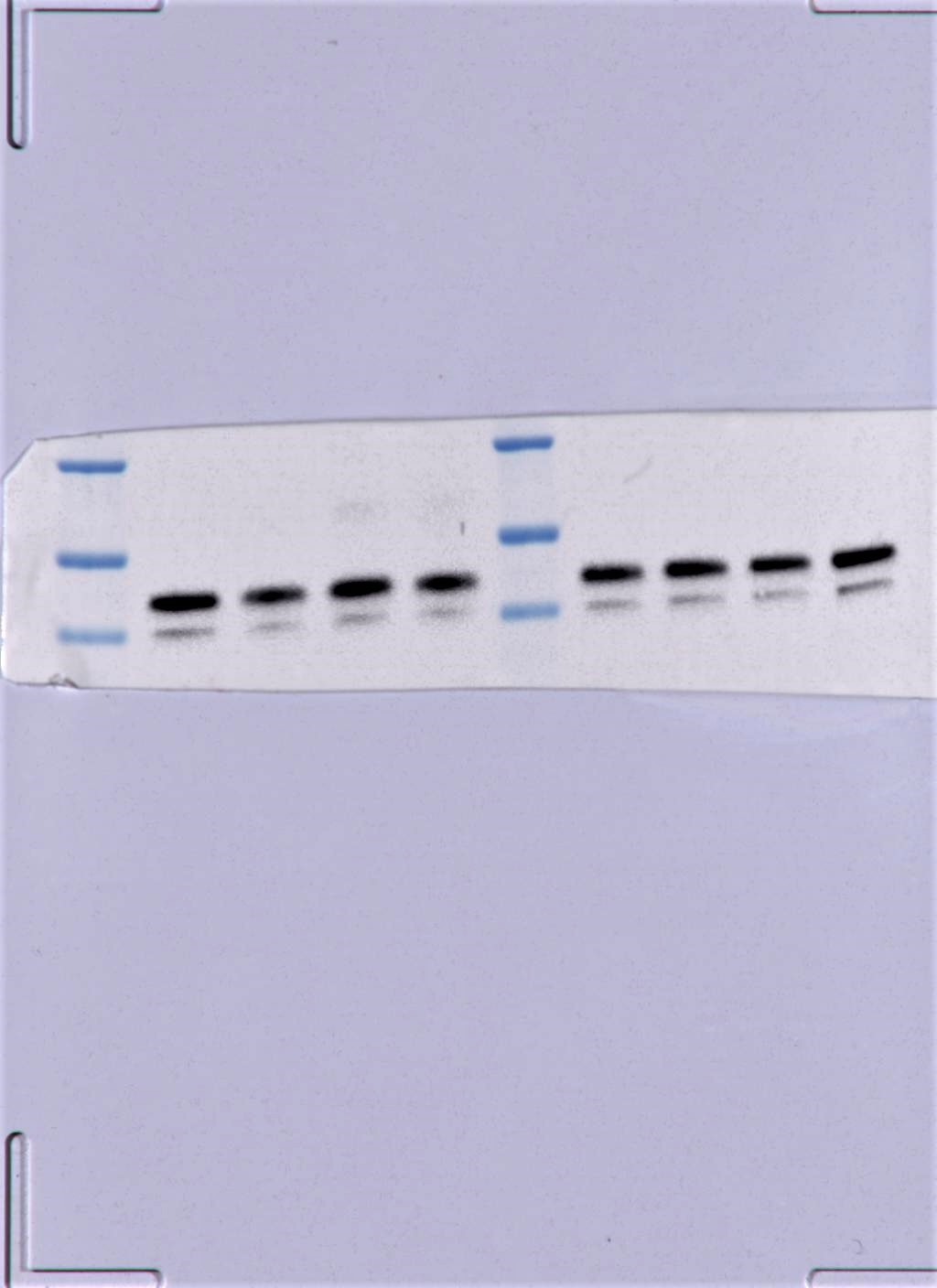

Supplement: Supplementary file 3 [file Image_2.jpeg]

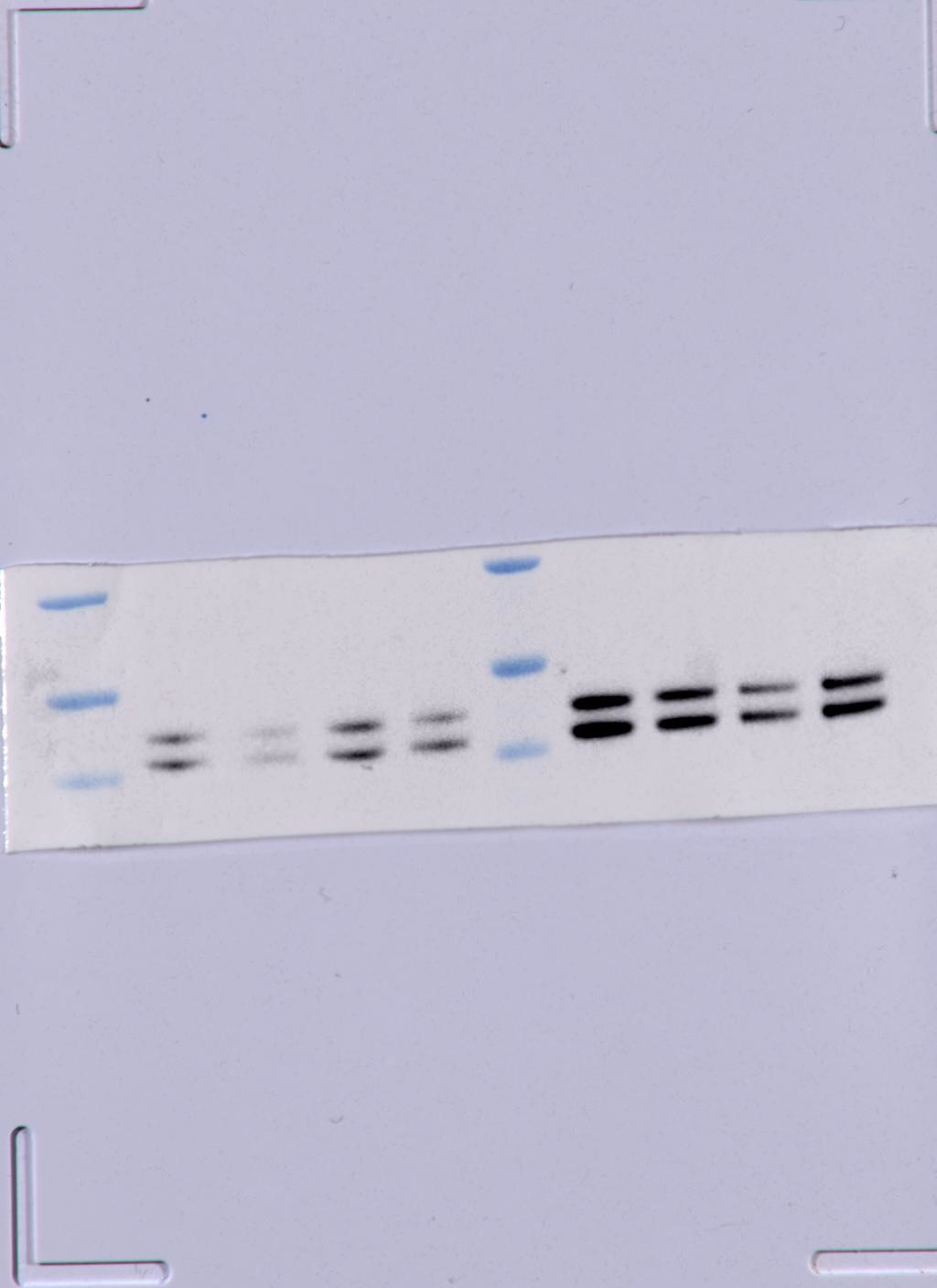

Supplement: Supplementary file 4 [file Image_3.jpeg]

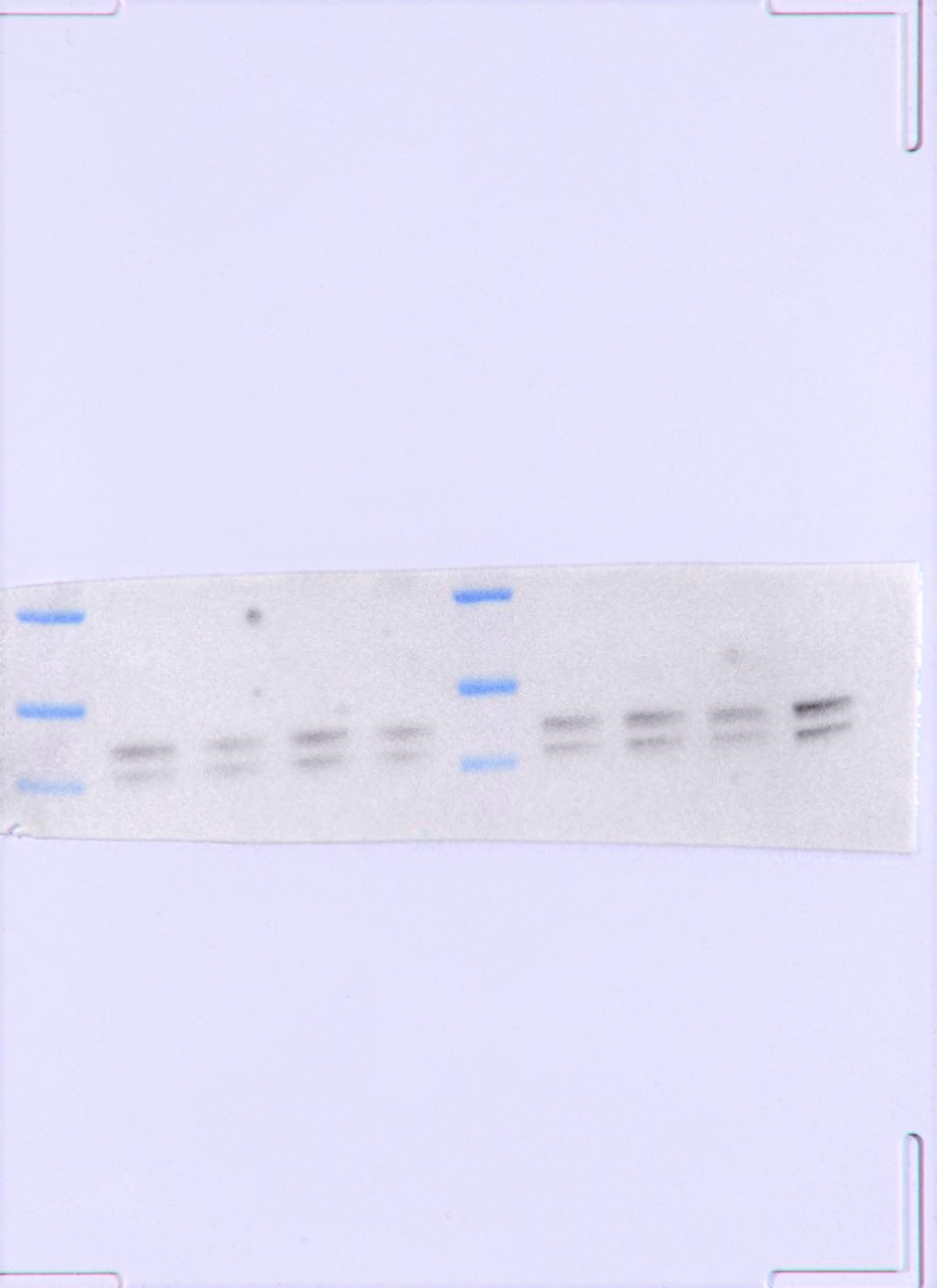

Supplement: Supplementary file 5 [file Image_4.jpeg]
